# MiR-144-3p inhibits gastric cancer progression and stemness via directly targeting GLI2 involved in hedgehog pathway

**DOI:** 10.1186/s12967-021-03093-w

**Published:** 2021-10-17

**Authors:** Yixun Lu, Benlong Zhang, Baohua Wang, Di Wu, Chuang Wang, Yunhe Gao, Wenquan Liang, Hongqing Xi, Xinxin Wang, Lin Chen

**Affiliations:** 1 Medical School of Chinese PLA, Beijing, 100853 China; 2 Department of General Surgery & Institute of General Surgery, The First Medical Center of Chinese PLA General Hospital, Beijing, 100853 China

**Keywords:** Gastric cancer, GLI2, MiR-144-3p, Cancer stem cells

## Abstract

**Background:**

Gastric cancer (GC) is the fifth most commonly diagnosed cancer worldwide. Due to the dismal prognosis, identifying novel therapeutic targets in GC is urgently needed. Evidences have shown that miRNAs played critical roles in the regulation of tumor initiation and progression. GLI family zinc finger 2 (GLI2) has been reported to be up-regulated and facilitate cancer progression in multiple malignancies. In this study, we focused on identifying GLI2-targeted miRNAs and clarifying the underlying mechanism in GC.

**Methods:**

Paired fresh gastric cancer tissues were collected from gastrectomy patients. GLI2 and miRNAs expression were detected in gastric cancer tissues and cell lines. Bioinformatics analysis was used to predict GLI2-targeted miRNAs and dual-luciferase reporter assay was applied for target verification. CCK-8, clone formation, transwell and flow cytometry were carried out to determine the proliferation, migration, invasion and cell cycle of gastric cancer cells. Tumorsphere formation assay and flow cytometry were performed to detail the stemness of gastric cancer stem cells (GCSCs). Xenograft models in nude mice were established to investigate the role of the miR-144-3p in vivo.

**Results:**

GLI2 was frequently upregulated in GC and indicated a poor survival. Meanwhile, miR-144-3p was downregulated and negatively correlated with GLI2 in GC. GLI2 was a direct target gene of miR-144-3p. MiR-144-3p overexpression inhibited proliferation, migration and invasion of gastric cancer cells. Enhanced miR-144-3p expression inhibited tumorsphere formation and CD44 expression of GCSCs. Restoration of GLI2 expression partly reversed the suppressive effect of miR-144-3p. Xenograft assay showed that miR-144-3p could inhibit the tumorigenesis of GC in vivo.

**Conclusions:**

MiR-144-3p was downregulated and served as an essential tumor suppressor in GC. Mechanistically, miR-144-3p inhibited gastric cancer progression and stemness by, at least in part, regulating GLI2 expression.

**Supplementary Information:**

The online version contains supplementary material available at 10.1186/s12967-021-03093-w.

## Background

Gastric cancer (GC) is the fifth most commonly diagnosed cancer and the fourth leading cause of cancer-related death in the world [1]. Due to dramatic advancement in Helicobacter pylori eradication, nutrition improvement, earlier diagnosis and therapeutic breakthroughs, the incidence of GC has declined in the past few decades. However, the prognosis of GC is still poor and disappointing [2]. Therefore, identifying novel therapeutic targets and clarifying the underlying mechanisms are urgently needed in GC.

MicroRNAs (miRNAs), a class of endogenous small non-coding RNAs, are short in length but powerful in regulation of gene expression [3]. More than 84% miRNAs down-regulate or silence the expression of target genes by directly binding to the 3’-UTR of mRNA [4]. Mountain studies have shown that miRNAs played critical roles in tumor proliferation, apoptosis, invasion, metastasis and drug resistance [5], etc. For example, miR-203 inhibits proliferation, migration and invasion of gastric cancer cells by downregulating Slug [6]; miR-1244 inhibits tumor metastasis by targeting FAK in intestinal-type gastric cancer [7]; miR-200c functions as a tumor suppressor in gastric cancer and overcomes drug resistance during cisplatin chemotherapy [8].

GLI family zinc finger 2, also known as GLI2, is one of the transcription factors in Hedgehog signaling pathway (HHSP). Previous studies showed that GLI2 was up-regulated and facilitated cancer progression in multiple malignancies, such as glioma [9], colorectal cancer [10] and bladder cancer [11]. In this paper, we showed that GLI2 was obviously upregulated in GC and high GLI2 expression indicated a poor survival. In addition, GLI2 is a critical messenger in crosstalk between PI3K/ Akt /mTOR pathway and HHSP. Increased PI3K/AKT/mTOR activity leads to enhanced hedgehog signaling via regulating GLI transcription factors [12]. Therefore, attempting to block or silence the expression of GLI2 will significantly help to suppress gastric cancer progression, and thus GLI2-targeted miRNA might be a good choice for gastric cancer treatment.

In this study, GLI2-targeted miRNA, miR-144-3p, was firstly identified in GC. The subsequent results illuminated that miR-144-3p inhibited gastric cancer progression in vitro and in vivo. What’s more, the study indicated that miR-144-3p attenuated the stemness of GCSCs. Finally, our results revealed that miR-144-3p inhibited gastric cancer progression and stemness via targeting GLI2.

## Methods

### Patients and clinical samples

Twenty-one pairs of fresh gastric adenocarcinoma (confirmed by preoperative biopsy and postoperative pathology) and adjacent normal tissues were collected from patients who undergone gastrectomy in Chinese PLA General Hospital from January 2020 to December 2020. Samples were collected immediately after tumor resection and kept in liquid nitrogen and then transferred to −80 °C refrigerator for preservation. All patients did not receive any adjuvant therapy (such as neoadjuvant chemoradiotherapy) before surgery. Written informed consents were obtained from all patients. This study was approved by the Medical Ethics Committee of Chines PLA General Hospital in accordance with the Declaration of Helsinki.

### Cell lines and cell culture

Human gastric cancer cell lines HGC-27, MKN-28, SGC-7901, MGC-803, BGC-823, NCI-N87 and immortalized gastric epithelial cell line GES-1 were purchased from the Cell Bank of Chinese Academy of Sciences (Shanghai, China). Adherent cells were cultured in Dulbecco’s modified Eagle’s medium (DMEM, GIBCO, NY, USA) with 10% fetal bovine serum (FBS) and 1% P/S in a standard condition at 37 °C with 5% CO_2_. Gastric cancer stem cells (GCSCs) were obtained and cultured as previously described [13, 14]. Briefly, GCSCs were extracted from fresh GC tissues resected in Chinese PLA General Hospital (PLAGH) and cultured in ultra-low-attachment 6-well plates (Corning, NY, USA) with modified DMEM/F12, 2% B27 supplements (Invitrogen, CA, USA), 1% ITS (insulin–transferrin–selenous acid, Corning, NY, USA), 20 ng/ml epidermal growth factor (Peprotech, Hartford, CT, USA), 10 ng/ml basic fibroblast growth factor (Peprotech), 10 ng/ml LIF (Peprotech) and 10 ng/ml gastrin I (Peprotech).

### RNA isolation and qRT-PCR

Total RNA from cell lines or tissues was extracted using TRIzol reagent according to the manufacturer’s instructions (Invitrogen). RNA quality and concentrations were determined by Nanodrop 2000 spectrophotometer (ThermoFisher, MA, USA). For mRNA quantification, cDNA was synthesized using a NovoScript^TM^ All-in-one 1st strand cDNA synthesis SuperMix with gDNA Purge, and qRT-PCR was performed using SYBR™ qPCR SuperMix Plus (Novoprotein, Shanghai, China). For miRNA, the Mir-X miRNA First-Strand Synthesis Kit and qRT-PCR TB Green Kit (Takara, Dalian, China) were used for quantification. All the primers used were listed in Additional file 1: Table S1. The expression levels of mRNA and miRNA were normalized to the expression of GAPDH and U6, respectively. The results were analyzed by the 2^−∆∆CT^ method and each sample was analyzed in triplicate.

### Oligonucleotides, plasmids and cell transfection

The miRNA mimics, inhibitors and negative controls were purchased from GenePharma (Shanghai, China). Gain or loss of function of miRNAs were accomplished by transfecting corresponding miRNA mimics or inhibitors, respectively. ΔN-GLI2 cDNA was subcloned into pCMV5 vector with endonucleases. ΔN-GLI2 mRNA, a truncated GLI2 version of GLI2 mRNA (the first 984 bases were deleted), can be translated into ΔN-GLI2 which is more transcriptionally active than full length GLI2 [15]. The GLI2 shRNA plasmid (target sequence: GCAACAAAGCCTTCTCCAA) was synthesized by GeneChem (Shanghai, China). The wild-type GLI2 3ʹ-UTR reporter plasmid containing miRNA binding sites and corresponding mutant-type reporter plasmid were purchased from Promega (#E2920, USA). The mimics, inhibitors and plasmids were transfected into cells by Lipofectamine 2000 reagent (Invitrogen). All the experiments were carried out accordance to the manufacturer’s instructions.

### Protein extraction and western blot

Cells were lysed on ice for 20 min using RIPA extraction buffer (Beyotime, Shanghai, China) containing protease inhibitor. Proteins were collected and the concentrations were determined by BCA protein kit (Solarbio, Beijing, China). Then, protein lysates were separated by SDS-PAGE and transferred to PVDF membrane (Millipore, Darmstadt, Germany). The membranes were blocked with 5% defatted milk for 1 h and incubated with primary antibodies overnight at 4 °C. Subsequently, the membranes were incubated with HRP-conjugated secondary antibodies at room temperature for 2 h. The β-Actin expression was used as internal control for normalization. The brands were detected by enhanced ECL chemiluminescence reagent (Beyotime). Details of the antibodies used in experiments were listed in Additional file 1: Table S2.

### Cell proliferation assay

The treated and control HGC-27 and SGC-7901 cells were examined by cell counting kit 8 (CCK-8) (Beyotime) to determined cell proliferation. Briefly, cells in logarithmic phase of each group were collected, resuspended, counted and then seeded at a density of 5000 cells per well into 96-well pates. Three wells were assigned for each group. CCK-8 assays were performed at 0, 24, 48, 72 and 96 h after cell culturation. That is, 10 μl CCK-8 reagent (Solarbio) was added to each well and absorbance at 450 nm was measured after the cells were incubated for 1 h at 37 °C. The proliferation curves were plotted to indicate cell viability.

### Colone formation assay

Cells of treatment and control groups were harvested and counted respectively. A total of 1000 cells were seeded per well in 6-well plates with complete culture medium. Cells were cultured at 37℃ with 5% CO_2_ for 10–14 days until visible colonies formed. Cells were fixed with 4% paraformaldehyde for 15 min and then stained with 0.1% crystal violet for 30 min. Colonies were photographed and counted.

### Cell cycle and flow cytometry analysis

Cells cultured in 6-well plates were trypsined, collected, centrifuged and the supernatant was removed. Then cells were washed with ice cold PBS and resuspended in 1 mL PBS. For the cell cycle, cells were fixed in 70% ethanol at 4 °C for 12–24 h. Subsequently, the cells were incubated with propidium staining solution with RNase A (Beyotime) at 37 °C for 30 min. The cell cycles were detected on BD flow cytometer (BD Biosciences, NJ, USA). For CD44 expression, cells were suspended in 80 μl binding buffer (Beyotime) and then incubated with 2 μl PE-Cy7 CD44 primary antibody (#560533, BD Biosciences) at 4 °C for 15–20 min in the dark. After centrifugation, the cells were resuspended with 500ul Hank’s Balanced Salt Solution (HBSS) and the expression of CD44 was detected on BD flow cytometer. Data analysis was carried out by FlowJo software (Version 10, Tree Star, USA).

### Transwell assay

The cell migration and invasion experiments were performed using 8 μm pore size transwell chambers (Corning) in 24-well plates. Cell medium containing 10% FBS was added to 24-well plates 0.6 ml per well. The 200 μl serum-free medium containing certain number of cells were added into each upper chamber. For migration, 4–5 × 10^4^ cells were diluted in 200 μl serum-free medium and added to each chamber. For invasion, the chambers were pre-coated with 50 μl diluted Matrigel (Corning, Matrigel: medium = 1:8), and then 8–10 × 10^4^ cells were diluted in 200 μl serum-free medium and added to each chamber. After incubation at 37 °C for 24 h, cells on the upper surface of chamber were swept by cotton swab slightly. Cells on the bottom of the chamber were fixed with paraformaldehyde for 15 min and stained with crystal violet for 30 min. Cells penetrated were imaged and counted at five random fields.

### Dual-luciferase reporter assay

Human embryonic kidney cell HEK293T was used for luciferase reporter assay. Once the cultured cells converged to 40–60%, 100 nM miR-144-3p mimics or negative controls (mimic-NC) were co-transfected with wild type (WT) or mutant type (MUT) GLI2 3’-UTR luciferase reporter plasmids respectively. After transfection for 48 h, a dual luciferase assay system (#E2920, Promega, WI, USA) was applied to determined relative luciferase activities according to the manufacturer’s instruction. The firefly luciferase activity was normalized to Renilla luciferase activity.

### Immunohistochemistry (IHC)

The paraffin-embedded tissues were cut into sections of 5 μm in thickness, then the sections were dewaxed and rehydrated. The slides were immersed in EDTA buffer (pH = 8.0) and heated in microwave oven with a specific temperature program for antigen retrieval. After rinsing with PBS, the slides were blocked with 3% BSA solution. Then the slides were incubated with primary antibody at 4 °C over night. Next, slides were incubated with HRP-coupled secondary antibodies at room temperature for 1 h and then 3,3-diaminobenzidine (DAB) was used for visualization of peroxidase reaction. Finally, the slides were re-stained with hematoxylin and photographed under microscope, and the images were analyzed.

### In-vivo xenograft assay

Five weeks old male BALB/c nude mice were purchased from Vital River Laboratory Animal Co. (Beijing, China) and raised in Specific Pathogen Free condition. Ten nude mice were randomly divided into negative control (NC) group or miR-144-3p mimic group (5 mice per group). Then, 1 × 10^6^ HGC-27 cells transfected with NC or mimics mixed with Matrigel (Corning) at a 1: 1 ratio. The xenograft model was established by injecting 200 μl abovementioned mixture into the right flanks of the nude mice subcutaneously. Tumor sizes were measured and recorded every 3–4 days with a vernier caliper. Three weeks later, the mice were euthanized. Tumors were dissected and measured. Tumor volume was calculated: V = 1/2×length×width^2^. All the animal experiments were performed in accordance with the guidelines approved by the Laboratory Animal Ethics Committee of Chinese PLA General Hospital.

### Bioinformatics analysis

GLI2-targeted miRNAs were predicted by online database TargetScan (http://www.targetscan.org/vert_72/), miRDB (http://mirdb.org/), miRanda (http://www.microrna.org/) and Starbase (http://starbase.sysu.edu.cn/index.php), respectively. The Venn diagram (https://bioinfogp.cnb.csic.es/tools/venny/) was used to intersect the predicted results. TCGA (https://www.cancer.gov/about-nci/organization/ccg/research/structural-genomics/tcga) and GEPIA (http://gepia.cancer-pku.cn/) database were used to analyze the GLI2 expression level in gastric cancer and adjacent normal tissues. The online database Kaplan–Meier plotter (http://kmplot.com/analysis/index.php) was applied to analyze survival outcomes. The expression of miRNAs and its correlation with target genes were analyzed based on TCGA database.

### Statistical analysis

SPSS Version 25.0 (IBM Corp, Armonk, NY, USA) was used for statistical analysis in this study. The results are expressed as mean ± standard deviation (SD). Student’s t-test (two-tailed) or analysis of variance (ANOVA) were applied to two or more groups comparison. The correlation between two parameters was examined by Pearson correlation test. GraphPad Prism 8 and ggplot2 package were used for data visualization. *p < 0.05, **p < 0.01, ***p < 0.001, ns = no significance. P < 0.05 was considered statistically significant.

## Results

### GLI2 is upregulated in gastric cancer and indicates poor survival

**Fig. 1:**
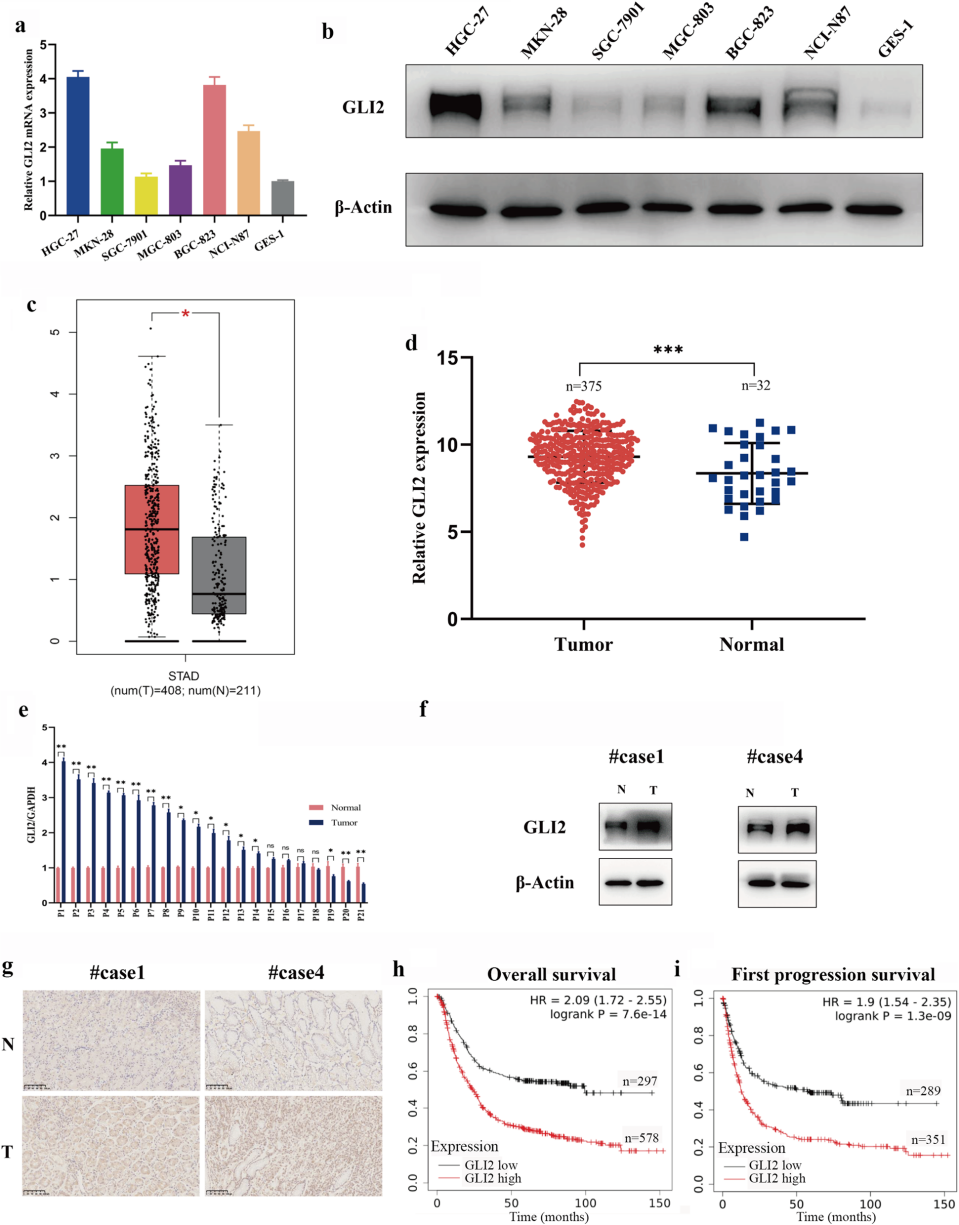
GLI2 is upregulated in gastric cancer and indicates a poor survival. **a**, **b** The mRNA and protein expression of GLI2 in six gastric cancer cell lines and GES-1. **c**, **d** GLI2 mRNA expression in GC in GEPIA and TCGA databases. **e** The relative GLI2 mRNA expression in 21 pairs of gastric cancer and adjacent normal tissues. **f**, **g** Protein expression and immunohistochemical staining of GLI2 in representative GC patients mentioned above. **h, i** Survival analysis based on K-M Plotter. (Scale bar = 100 μm, *p < 0.05, **p < 0.01, ***p < 0.001, ns = no significance)

According to recent studies, GLI2 is up-regulated in multiple cancers [9–11]. In order to determine the expression of GLI2 in gastric cancer, we firstly detected the GLI2 mRNA in 6 gastric cancer cell lines and immortalized gastric epithelial cell line GES-1. Through qRT-PCR, we found that GLI2 was up-regulated in most gastric cancer cell lines compared with GES-1 (Fig. 1a). Then, we further detected the expression of GLI2 protein in GC. In accordance with qRT-PCR, Western blot (WB) results showed that GLI2 protein was highly expressed in most gastric cancer cell lines, especially in HGC-27 and BGC-823 (Fig. 1b). Next, we explored the expression of GLI2 mRNA in gastric cancer tissues through GEPIA and TCGA databases. As is shown in Fig. 1c, d, GLI2 expression was up-regulated in gastric cancer compared with adjacent normal tissues, and the differences were both statistically significant. Subsequently, we detected GLI2 mRNA levels in 21 paired gastric adenocarcinoma and adjacent normal tissues from GC patients and found that GLI2 was highly expressed in 14/21 patients (Fig. 1e). What’s more, WB and IHC showed the protein expression of GLI2 in representative patients (Fig. 1f, g). In addition, based on K-M Plotter database, patients with higher GLI2 expression harvested a lower overall survival (OS) and first progression survival (FPS), indicating that high GLI2 expression predicted poor prognosis in GC (Fig. 1h, i). Collectively, GLI2 was highly expressed in GC and indicated a poor survival.

### MiR-144-3p downregulates GLI2 expression in gastric cancer

**Fig. 2:**
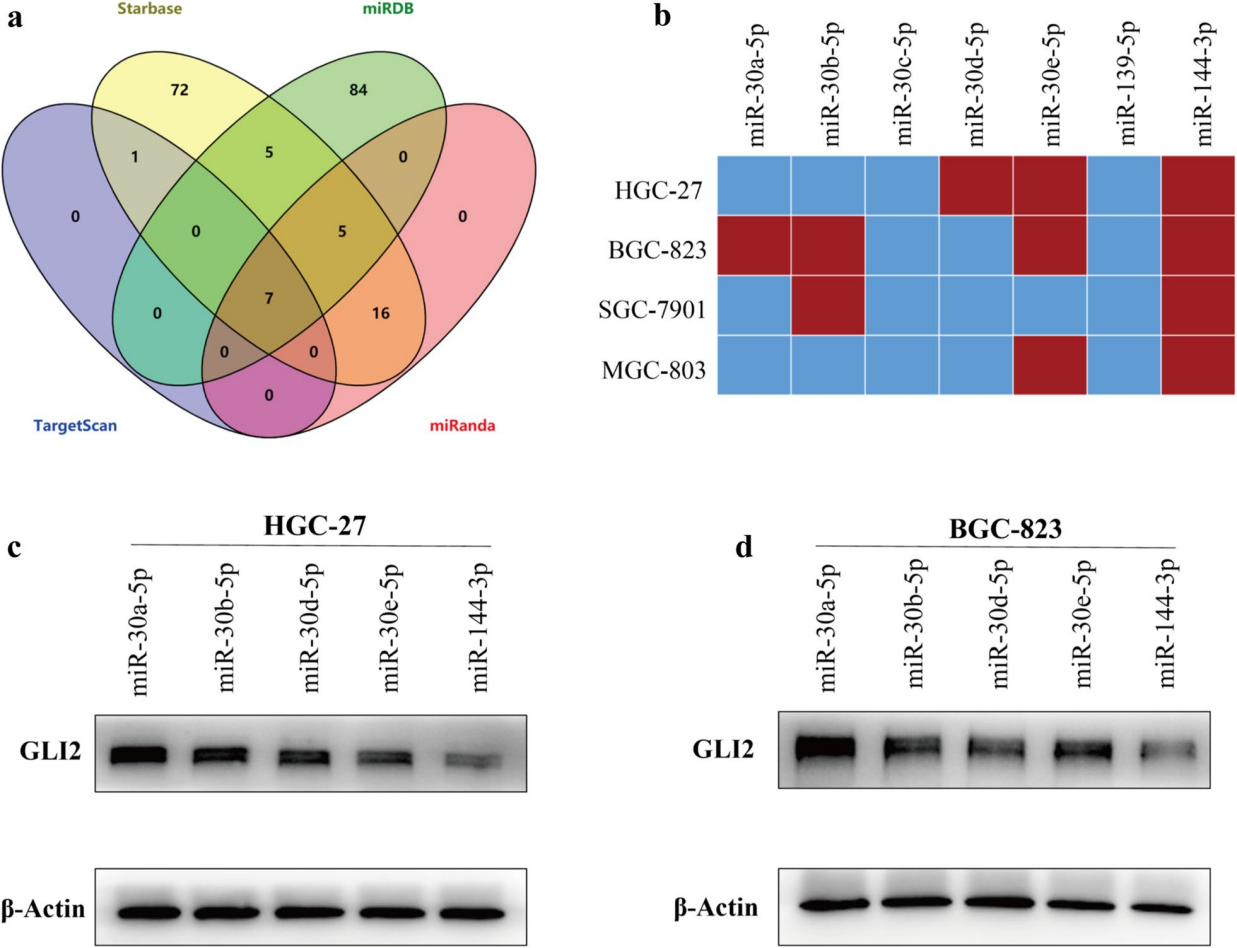
MiR-144-3p downregulates GLI2 expression in gastric cancer. **a** The venn diagram of GLI2-targeted miRNAs predicted by bioinformatic analysis. **b** The heatmap was drawn to exhibit the decrease extent of GLI2 mRNA (red ≥ 25%, blue < 25%) in four gastric cancer lines after transfection with different kinds of miRNA mimics. **c**, **d** The protein expression of GLI2 in HGC-27 and BGC-823 after transfection with different kinds of miRNA mimics

Considerable studies have shown that miRNAs could modulate gene expression [3, 4, 16]. In order to identify the potential GLI2-targeted miRNAs in GC, online databases including TargetScan, miRDB, miRanda and Starbase were used for bioinformatic prediction. As a result, seven candidate miRNAs (miR-30a-5p, miR-30b-5p, miR-30c-5p, miR-30d-5p, miR-30e-5p, miR-139-5p and miR-144-3p) were obtained when we intersected the predicted results (Fig. 2a). However, among these candidates, we were curious that who was the real player in GC. Therefore, we synthesized the miRNA mimics of these seven candidates and transferred them into four gastric cancer cell lines (HGC-27, BGC-823, SGC-7901, and MGC-803) respectively, and the changes of GLI2 mRNA were detected by qRT-PCR. A heatmap was drawn based on whether the decrease of GLI2 mRNA was greater than 25% (Fig. 2b). The results showed that five miRNA mimics down-regulated GLI2 mRNA expression in at least one gastric cancer cell line, while miR-144-3p down-regulated GLI2 in all the four gastric cancer cell lines (Fig. 2b). Subsequently, the above five kinds of miRNA mimics were transfected into HGC-27 and BGC-823 cell lines, and the changes of GLI2 protein were detected by WB. Consistent with the foregoing results, the GLI2 protein expression decreased most significantly after transfection with miR-144-3p in both GC cell lines (Fig. 2c, d). In short, the above results suggested that miR-144-3p could down-regulate GLI2 expression in GC.

### MiR-144-3p is downregulated and negatively correlated with GLI2 expression in gastric cancer

**Fig. 3:**
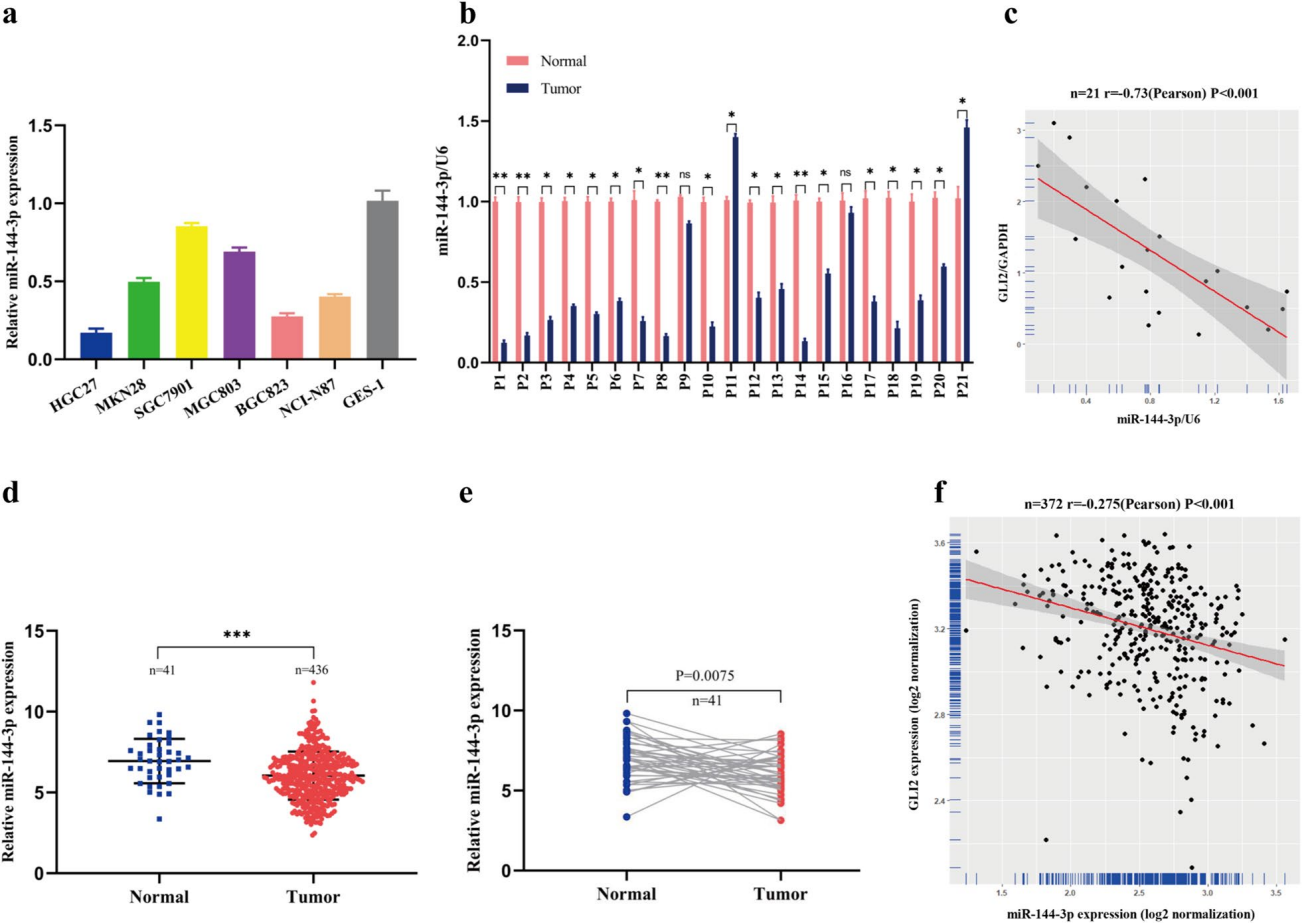
MiR-144-3p is downregulated and negatively correlated with GLI2 expression in gastric cancer. **a** The relative miR-144-3p expression in six gastric cancer cell lines and GES-1. **b** The relative miR-144-3p expression in paired tissues of 21 GC patients. **c** The correlation of miR-144-3p and GLI2 mRNA expression in gastric cancer tissues of the 21 patients. **d** The relative expression of miR-144-3p in gastric cancer and normal tissues in TCGA. **e** The relative expression of miR-144-3p in paired cases of gastric cancer and adjacent normal tissues in TCGA (n = 41). **f** The correlation of miR-144-3p and GLI2 mRNA expression in gastric cancer tissues of TCGA (n = 372). (*p < 0.05, **p < 0.01, ***p < 0.001, ns = no significance)

Based on the results mentioned above, we wondered the expression profile of miR-144-3p and its correlation with GLI2 mRNA in GC. Firstly, we quantified the expression level of miR-144-3p in gastric cancer cell lines and GES-1 by qRT-PCR. The results showed that miR-144-3p was down-regulated in most gastric cancer cell lines compared with GES-1 (Fig. 3a). In addition, we detected the relative expression of miR-144-3p in paired gastric cancer and adjacent normal tissues of 21 patients, and the results showed that miR-144-3p was frequently down-regulated in gastric cancer tissues (17/21) (Fig. 3b). What’s more, the correlation between miR-144-3p and GLI2 mRNA expression was analyzed in the gastric cancer tissues of the 21 patients. The results showed a significant negative correlation between miR-144-3p and GLI2 mRNA (Pearson r = − 0.73, p < 0.001) (Fig. 3c). Next, based on the STAD project of TCGA, we analyzed the relative expression of miR-144-3p in gastric cancer and adjacent normal tissues. Similarly, miR-144-3p expression was significantly down-regulated in gastric cancer tissues (p < 0.001) (Fig. 3d). The data of 41 paired cases of gastric cancer and adjacent normal tissues in TCGA also showed that miR-144-3p was low expressed in cancer tissues (p = 0.0075) (Fig. 3e). Moreover, there was a significant negative correlation between miR-144-3p and GLI2 mRNA expression in 372 gastric cancer samples in TCGA (Pearson r = − 0.275, p < 0.001) (Fig. 3f). Generally, these results showed that miR-144-3p expression was downregulated and negatively correlated with GLI2 mRNA in GC.

### MiR-144-3p directly targets 3ʹ-UTR of GLI2 mRNA

**Fig. 4:**
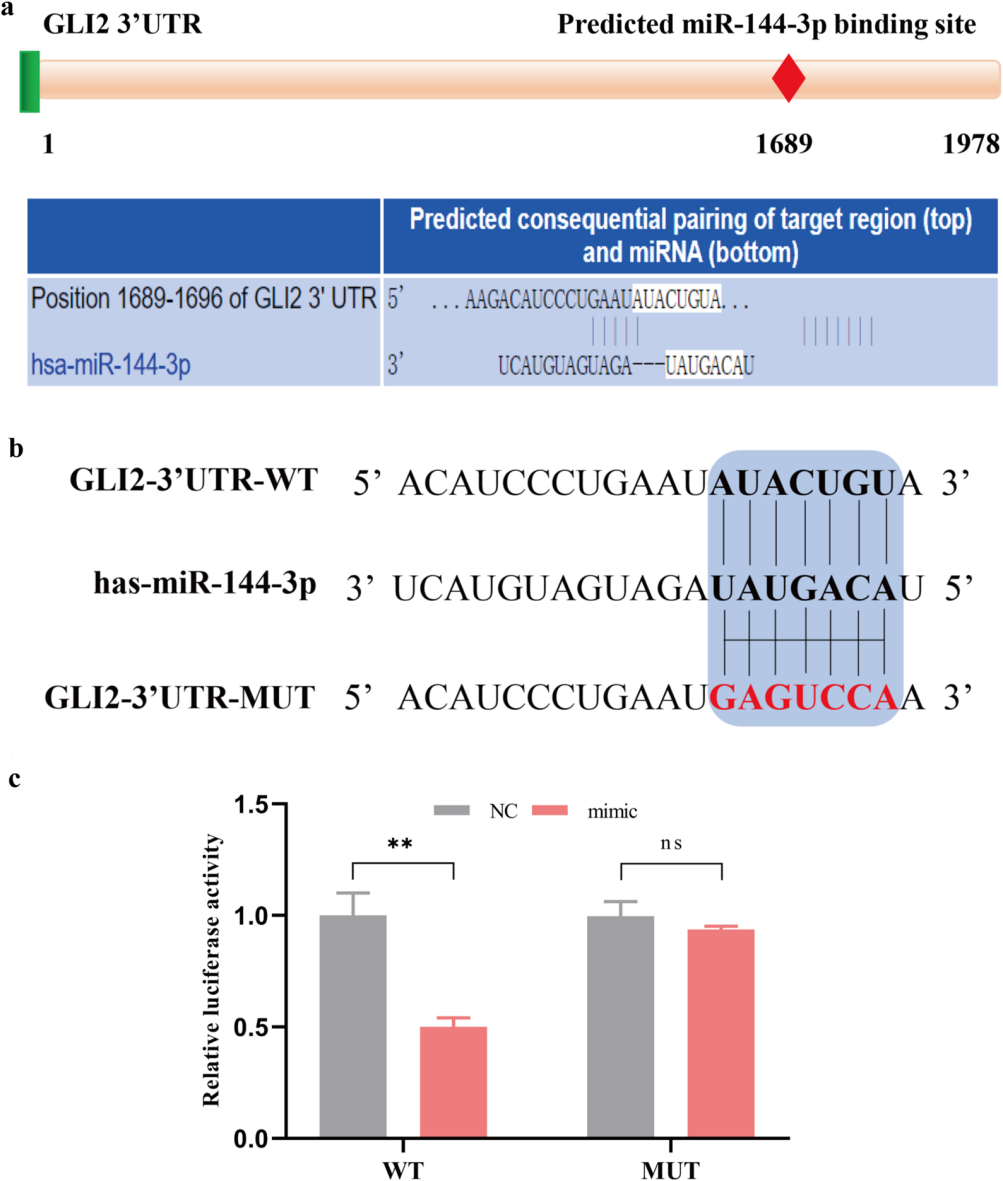
MiR-144-3p directly targets 3’-UTR of GLI2 mRNA. **a** The potential miR-144-3p binding sites in 3’-UTR of GLI2 mRNA predicted by TargetScan. **b** The sequence of the WT-GLI2 and MUT-GLI2 3’-UTR reporter plasmids. **c** The relative luciferase activity in HEK293T co-transfected with miR-144-3p mimics or negative control (NC) and WT-GLI2 or MUT-GLI2 reporter plasmid. (**p < 0.01, ns = no significance)

Based on the previous results of the study, we speculated that GLI2 mRNA was a direct target gene of miR-144-3p. To verify this hypothesis, bioinformatics analysis was applied to predict the miR-144-3p binding sites of GLI2 mRNA 3ʹ-UTR. According to TargetScan, the 1689–1696 sequences of GLI2 mRNA 3ʹ-UTR were the potential binding sites of miR-144-3p (Fig. 4a). Therefore, the wild-type (WT) and mutant-type (MUT) plasmids containing miR-144-3p binding sites were constructed (Fig. 4b), and the dual-luciferase reporter assay was performed in HEK293T. The results showed that the WT-GLI2 luciferase activity was significantly decreased when co-transfected with miR-144-3p mimics (p < 0.01). However, there was no significant change of MUT-GLI2 luciferase activity after co-transfection with miR-144-3p mimics (Fig. 4c). Collectively, the above results indicated that GLI2 was a direct target gene of miR-144-3p.

### MiR-144-3p inhibits gastric cancer progression and stemness

**Fig. 5:**
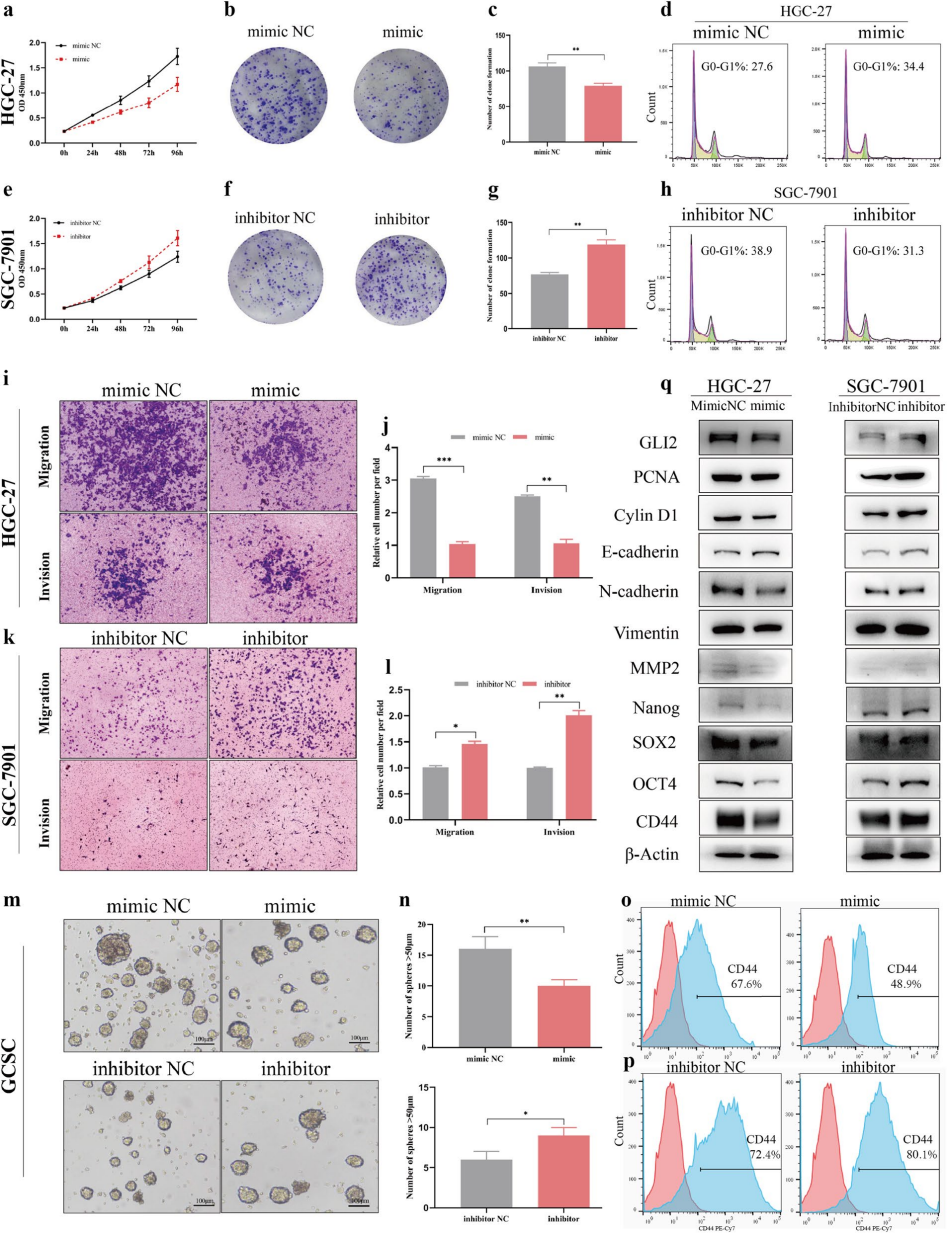
MiR-144-3p inhibits gastric cancer progression and stemness. **a** CCK-8 assay to detect cell viability of HGC-27 transfected with miR-144-3p mimics or negative control (NC). **b**, **c** Clone formation assay of HGC-27 transfected with miR-144-3p mimics or NC. The clone number of HGC-27 was counted. **d** Cell cycle was detected by flow cytometry in HGC-27. **e** Cell proliferation of SGC-7901 was detected by CCK-8 after transfection with miR-144-3p inhibitor or NC. **f**, **g** Clone formation assay of SGC-7901 transfected with miR-144-3p inhibitor or NC. The clone number of SGC-7901 was counted. **h** Cell cycle was detected by flow cytometry in SGC-7901. **i, j** Transwell assay to detect the migration and invasion of HGC-27 treated with mimics or NC. The migrated and invaded cells were counted. **k**, **l** Transwell assay to detect migration and invasion of SGC-7901 treated with inhibitor or NC. The migrated and invaded cells were counted. **m**, **n** Tumorsphere formation assay of gastric cancer stem cells (GCSCs) treated with miR-144-3p mimic or inhibitor and corresponding NC. The number of tumorsphere > 50 μm in per field was counted. **o**, **p** The CD44 expression of GCSCs treated with miR-144-3p mimic or inhibitor was detected by flow cytometry. **q** The related protein expression after miR-144-3p intervention in HGC-27 and SGC-7901. (Scale bar = 100 μm, *p < 0.05, **p < 0.01, ***p < 0.001)

Previous studies have shown that miR-144-3p was a critical tumor suppressor in multiple cancers including colorectal cancer [17], hepatocellular carcinoma [18], cholangiocarcinoma [19] etc. However, the function and mechanism of miR-144-3p in GC have not been thoroughly explored. In the following study, we chose to transfect miR-144-3p mimics into HGC-27 and miR-144-3p inhibitor into SGC-7901 to explore the function of miR-144-3p in GC. Firstly, we manipulated expression of miR-144-3p to test its effect on the proliferation ability of gastric cancer cells. After transfection with miR-144-3p mimics, the proliferation ability and the clone formation rate of GC cells were decreased (Fig. 5a–c), while the number of GC cells in G0-G1 phase was increased (Fig. 5d). On the contrary, cell proliferation ability and the rate of clone formation were enhanced after transfection with miR-144-3p inhibitor (Fig. 5e–g), while the number of GC cells in G0-G1 phase was decreased (Fig. 5h). Subsequently, we explored the effect of miR-144-3p abnormal expression on invasion and migration of gastric cancer cells. The results showed that the migration and invasion ability was significantly weakened after transfection with miR-144-3p mimics (Fig. 5i, j) while cell motility was enhanced after transfection with miR-144-3p inhibitor (Fig. 5k, l). In addition, previous evidences suggested that HHSP played an important role in maintenance of cancer cell stemness. Therefore, we speculated that abnormal expressed miR-144-3p could exert an influence on the stemness of GC cells. Then GCSCs were cultured, and miR-144-3p mimics or inhibitor were transfected. As expected, the results showed that after transfected miR-144-3p mimics the tumorsphere formation ability of GCSCs was weakened and CD44 expression was decreased significantly. However, the tumorsphere formation ability was enhanced and CD44 expression was elevated after transfection with miR-144-3p inhibitor (Fig. 5m–p). Finally, we examined the changes of hedgehog target gene (Cyclin D1) [20], proliferation, invasion, epithelial-mesenchymal transformation (EMT) and stemness related proteins by WB in HGC-27 and SGC-7901 after transfection with miR-144-3p mimics or inhibitor respectively. The results indicated that miR-144-3p inhibited proliferation, invasion, EMT and attenuated the stemness of gastric cancer cells (Fig. 5q). Taken together, these results showed that enhanced miR-144-3p expression inhibited gastric cancer progression and stemness.

### GLI2 reverses miR-144-3p-mediated suppression in gastric cancer

**Fig. 6:**
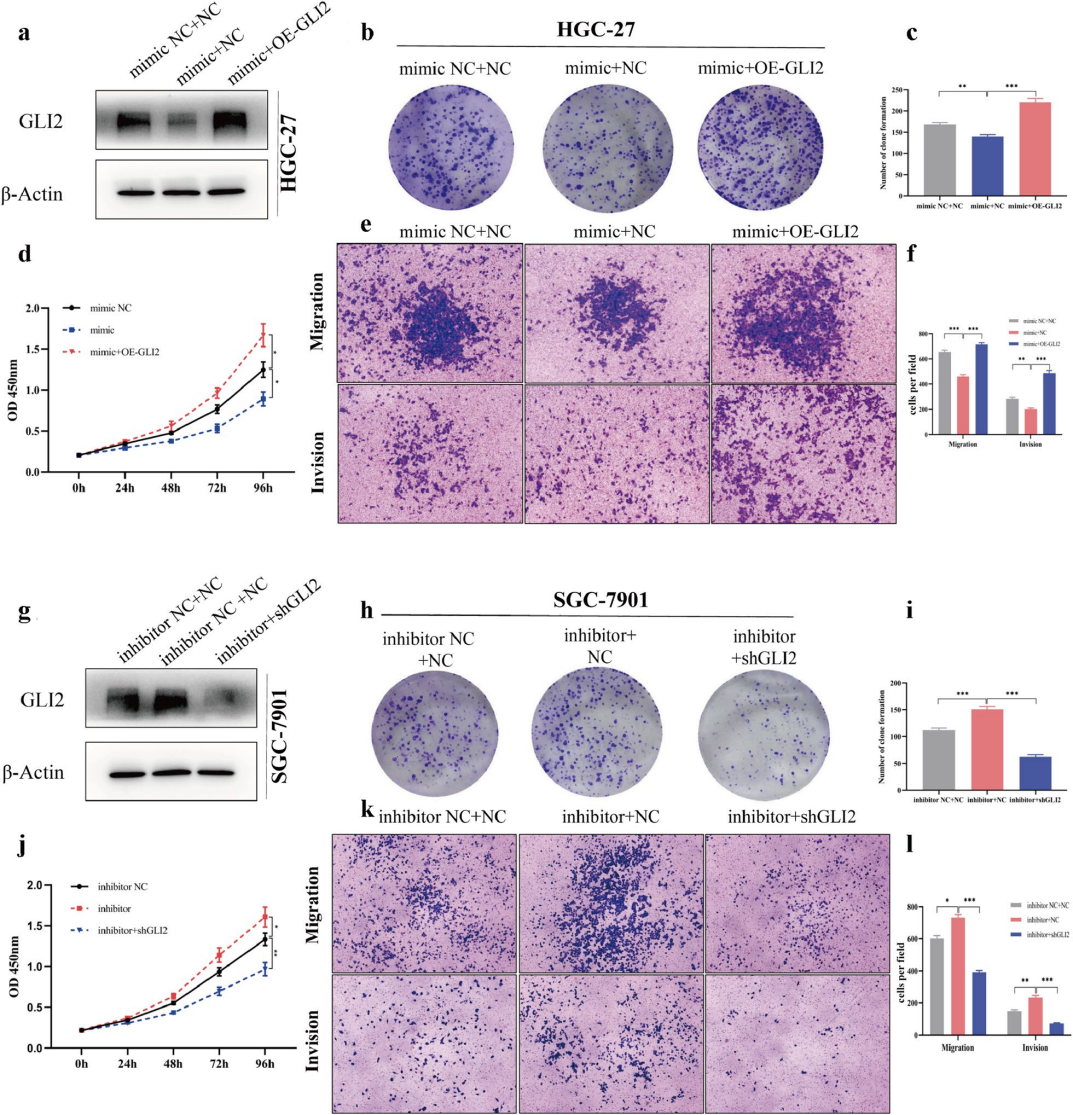
GLI2 reverses miR-144-3p-mediated suppression in gastric cancer. **a** The protein expression of GLI2 in HGC-27 that co-transfected miR-144-3p mimics and GLI2 overexpression (OE-GLI2) plasmid. **b**, **c** Clone formation of HGC-27 and clone number was counted. **d** The proliferation of HGC-27 was determined by CCK-8 assay. **e**, **f** Migration and invasion of HGC-27 was detected by Transwell assay and the migrated and invaded cells were counted. **g** The protein expression of GLI2 in SGC-7901 that co-transfected miR-144-3p inhibitor and GLI2 shRNA (shGLI2) plasmid. **h**, **i** Clone formation of SGC-7901 and clone number was counted. **j** The proliferation of SGC-7901 was determined by CCK-8 assay. **k**, **l** Transwell assay was performed to detect the migration and invasion of HGC-27 and the cells were counted. (*p < 0.05, **p < 0.01, ***p < 0.001)

The former parts of this study conformed that miR-144-3p down-regulates the GLI2 expression and inhibits the progression of gastric cancer. Nevertheless, it was still unclear that whether miR-144-3p inhibited gastric cancer progression via regulating GLI2. In order to clarify it, rescue experiments were conducted. Firstly, we restored the expression of GLI2 in HGC-27 transfected with miR-144-3p mimics, and the results showed that the tumor suppressive effects of miR-144-3p were reversed (Fig. 6a–f). Subsequently, we knocked down GLI2 expression in SGC-7901 transfected with miR-144-3p inhibitor, and the results showed that knockdown of GLI2 offset the cancer-promoting effect of miR-144-3p inhibitor (Fig. 6g–l). These results showed that miR-144-3p inhibited gastric cancer progression by, at least in part, regulating the expression of GLI2.

### MiR‑144-3p inhibits tumorigenesis in vivo

**Fig. 7:**
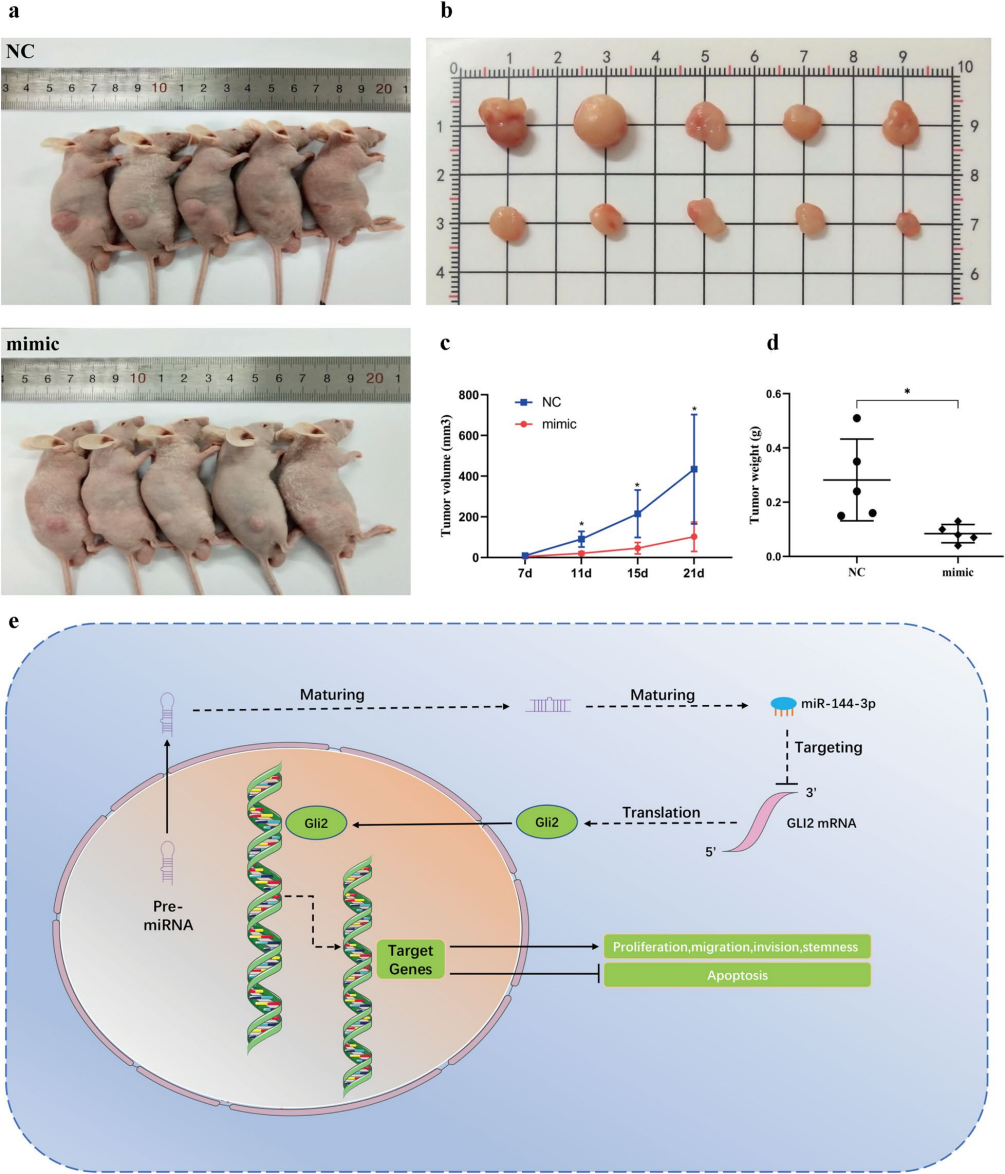
MiR‑144-3p inhibits tumorigenesis in vivo. **a** Images of nude mice three weeks after xenograft transplantation. **b** Images of tumors three weeks after xenograft transplantation. **c** Tumor volume was recorded and statistically analyzed during the 3 weeks. **d** Tumor weight was measured and statistically analyzed after mice sacrifice. **e** The schematic mechanism of miR-144-3p inhibiting gastric cancer progression and stemness via regulating GLI2 expression. (*p < 0.05)

In order to further investigate whether miR-144-3p could inhibit tumor growth in vivo, HGC-27 cells transfected with miR-144-3p NC or miR-144-3p mimics were injected into male BALB/c nude mice subcutaneously (Fig. 7a). Three weeks later, the mice were sacrificed. Tumors were isolated (Fig. 7b), and the volume and weight were measured. The results showed that the tumor volume ([102 ± 72.6] mm^3^ vs [434 ± 268.8] mm^3^, p < 0.05) and tumor weight ([0.084 ± 0.034] g vs [0.282 ± 0.15] g, p < 0.05) of miR-144-3p mimics group were significantly lower than those of miR-144-3p NC group (Fig. 7c, d). Obviously, the results showed that miR‑144-3p could serve as a tumor suppressor and inhibit tumorigenesis in vivo. Taken together, our studies indicated that miR‑144-3p inhibited gastric cancer progression and stemness by regulating GLI2 expression (Fig. 7e).

## Discussion

Hedgehog signaling pathway (HHSP), a stemness related and highly conserved pathway, is usually activated during embryonic development and inactive or extremely low expressed in adults [21]. Previous studies have shown that the activation of HHSP was closely related to the initiation and progression of multiple malignant tumors [22–24]. GLI2, an end effector of the HHSP, has been reported to be up-regulated and serve as a cancer-promoting gene in several malignant tumors including glioma [9], colon cancer [10], bladder cancer [11], etc. In this study, we showed that GLI2 was frequently up-regulated in GC and high GLI2 expression indicated a poor survival. Although studies have shown that GLI inhibitor GANT61 could inhibit cancer progression in vivo and vitro [25], there is still a long way from clinical application.

Recently, mountain evidences have shown that miRNAs played pivotal roles in cancer regulation [3, 5]. In fact, more than 84% miRNAs can silence or inhibit the expression of target genes [26]. What’s more, several miRNA-based next generation drugs have exhibited promising therapeutic landscape in cancers [11, 27]. Undoubtedly, therapeutic miRNA or RNAi is an effective method of cancer treatment. Therefore, we wondered that whether there were miRNAs which directly target GLI2 and thus inhibited the progression of gastric cancer. However, no relevant studies have been reported in GC.

In this study, we first identified GLI2-targeted miRNAs through bioinformatic analysis. For the seven candidate miRNAs obtained, we further showed that miR-144-3p could downregulate GLI2 expression most significantly. What's more, we discovered that miR-144-3p was significantly down-regulated in gastric cancer cell lines and tissues. In addition, there was a significantly negative correlation between the expression of miR-144-3p and GLI2 mRNA. Therefore, we speculated that miR-144-3p was a tumor suppressor in GC. Then, the dual-luciferase assay confirmed that miR-144-3p can directly bind to the 3ʹ-UTR of GLI2 mRNA. Briefly, our study revealed for the first time that GLI2 was a direct target gene of miR-144-3p. MiR-144-3p, 20nt in length, is a kind of small non-coding RNA derived from chromosome 17q11.2. Most studies reported that miR-144-3p served as a tumor suppressor in various kinds of cancers such as colorectal cancer [17], hepatocellular carcinoma [18], cholangiocarcinoma [19], oral squamous cell carcinoma [28], cervical cancer [29], prostate cancer [30], glioma [31] etc. However, miR-144-3p has also been reported to promote the progression of nasopharyngeal carcinoma [32] and papillary thyroid carcinoma [33]. Meanwhile, the role of miR-144-3p in GC and the underlying mechanism have not been thoroughly explored. In the present study, we revealed that overexpression miR-144-3p inhibited proliferation, migration and invasion of gastric cancer cells. In terms of mechanism, rescue experiments proved that miR-144-3p inhibited gastric cancer progression by, at least in part, regulating GLI2 expression.

Cancer stem cells (CSCs), possessing the ability to initiate tumor growth and sustain self-renewal as well as differentiation, are considered to be responsible for the heterogeneity, drug resistance, metastasis and recurrence of gastric cancer [34–36]. Our previous study also showed that the HHSP played a crucial role in stemness maintenance of GCSCs [37]. Therefore, we speculated that abnormally expressed miR-144-3p could exert influence on the stemness of GCSCs by regulating the expression of GLI2. As expected, both tumorsphere formation assay, CD44 expression detected by flow cytometry and stemness related markers (SOX-2, OCT-4, Nanog) detected by WB confirmed that miR-144-3p could attenuate the stemness of gastric cancer cells. In addition, epithelial mesenchymal transformation (EMT) is deeply involved in tumor initiation, invasion and metastasis. Our results also showed that miR-144-3p inhibited EMT in GC, which was in accordance with Liu’s results performed in renal cell carcinoma [38]. Finally, xenograft assay in nude mice further confirmed the tumor suppressor role of miR-144-3p in vivo.

## Conclusions

In conclusion, our results indicated that miR-144-3p served as an essential tumor suppressor in gastric cancer. Mechanistically, miR-144-3p inhibited gastric cancer progression and stemness via regulating GLI2 expression. Therefore, manipulation of miR-144-3p expression might provide a promising therapeutic strategy for gastric cancer. However, further studies are expected to detail the role of miR-144-3p/hedgehog/GLI2 axis in GC.

## Supplementary Information

**Additional file 1: Table S1.** All primers used in experiments. **Table S2.** Details of antibodies used in experiments.

## Data Availability

The datasets used and/or analysed during the current study are available from the corresponding author on reasonable request.

## Abbreviations

GC: Gastric cancer
GLI2: GLI family zinc finger 2
GCSCs: Gastric cancer stem cells
HHSP: Hedgehog signalling pathway
PBS: Phosphate buffer saline
FBS: Fetal bovine serum
P/S: Penicillin/streptomycin
LIF: Leukemia inhibitory factor
HBSS: Hank’s balanced salt solution
qRT-PCR: Quantitative reverse transcription PCR
IHC: Immunohistochemistry
shRNA: Short hairpin RNA
TCGA: The cancer genome atlas
EMT: Epithelial–mesenchymal transformation
