# Supplementary material for: MiR-144-3p inhibits gastric cancer progression and stemness via directly targeting GLI2 involved in hedgehog pathway
Source: J Transl Med. 2021 Oct 17;19:432. doi: 10.1186/s12967-021-03093-w (PMC8521984; doi:10.1186/s12967-021-03093-w)
Supplement: Supplementary file 1 — Additional file 1: Table S1. All primers used in experiments. Table S2. Details of antibodies used in experiments. [file 12967_2021_3093_MOESM1_ESM.docx]

**Table S1 All primers used in experiments**

| **Gene** | **Forward** **(5’-3’)** | **Reverse (5’-3’)** |
| --- | --- | --- |
| GLI2 | \| AGGGATGACTGTAAGCAGGAGG \| \| --- \| | TGGATGTGCTCGTTGTTGATG |
| GAPDH | GGAAGCTTGTCATCAATGGAAATC | TGATGACCCTTTTGGCTCCC |
| U6 | CTCGCTTCGGCAGCACA | AACGCTTCACGAATTTGCGT |
| miR-30a-5p | TGTAAACATCCTCGACTGGAAG | Universal Adaptor Reverse Primer |
| miR-30b-5p | TGTAAACATCCTACACTCAGCT | Universal Adaptor Reverse Primer |
| miR-30c-5p | TGTAAACATCCTACACTCTCAGC | Universal Adaptor Reverse Primer |
| miR-30d-5p | TGTAAACATTCCCCGACTGGAAG | Universal Adaptor Reverse Primer |
| miR-30e-5p | TGTAAACATCCTTGACTGGAAG | Universal Adaptor Reverse Primer |
| miR-139-5p | TCTACAGTGCACGTGTCTCCAGT | Universal Adaptor Reverse Primer |
| miR-144-3p | TACAGTATAGATGATGTACT | Universal Adaptor Reverse Primer |

**Table S2 Details of antibodies used in experiments**

| **Antibody** | **WB/ IHC Dilution** | **Specificity** | **Company** | **Category Number** |
| --- | --- | --- | --- | --- |
| β-Actin | 1:1000 | Mouse monoclonal | CST | #3700 |
| GLI2 | 1 :1000 (WB) | Rabbit monoclonal | CST | #2585 |
| GLI2 | 1:400(IHC) | Rabbit polyclonal | BOSTER | # A00701-5 |
| PCNA | 1:1000 | Rabbit monoclonal | CST | # 13110 |
| Cyclin D1 | 1:1000 | Rabbit monoclonal | CST | # 55506 |
| E-cadherin | 1:1000 | Mouse monoclonal | CST | # 14472 |
| N-cadherin | 1:1000 | Rabbit monoclonal | CST | # 13116 |
| Vimentin | 1:1000 | Rabbit monoclonal | CST | # 5741 |
| MMP-2 | 1:1000 | Rabbit monoclonal | CST | # 40994 |
| Nanog | 1:2000 | Rabbit monoclonal | CST | #4903 |
| SOX2 | 1:1000 | Rabbit monoclonal | CST | #3579 |
| OCT4 | 1:1000 | Rabbit antibody | CST | # 2750 |
| CD44 | 1:1000 | Mouse monoclonal | CST | # 3570 |
| IgG (HRP-linked) | 1:3000 | Rabbit monoclonal | CST | # 7074 # 7076 |
